# Prevalence and Associated Factors of Problematic Use of Smartphones Among Adults in Qassim, Saudi Arabia: Cross-sectional Survey

**DOI:** 10.2196/37451

**Published:** 2022-05-23

**Authors:** Abdulrahman Al-Mohaimeed, Mansour Alharbi, Ilias Mahmud

**Affiliations:** 1 Department of Family and Community Medicine College of Medicine Qassim University Buraidah Saudi Arabia; 2 Department of Psychiatry College of Medicine Qassim University Buraidah Saudi Arabia; 3 Department of Public Health College of Public Health and Health Informatics Qassim University Al Bukairiyah Saudi Arabia

**Keywords:** smartphone, smartphone addiction, problematic use of smartphones, mobile phone dependence, problematic use of mobile phones, Saudi Arabia, addiction, psychosocial, cross-sectional survey, psychological health, student, mental health

## Abstract

**Background:**

The Kingdom of Saudi Arabia (KSA) ranks third globally in smartphone use. Smartphones have made many aspects of life easier. However, the overuse of smartphones is associated with physical and psychosocial problems.

**Objective:**

The aim of this paper is to estimate the prevalence and associated factors of problematic use of smartphones among adults in the Qassim region of KSA.

**Methods:**

We enrolled 715 participants using cluster random sampling for this cross-sectional survey. We assessed the problematic use of smartphones using the short version of the Smartphone Addiction Scale.

**Results:**

We estimated the prevalence of problematic smartphone use among adults at 64% (453/708). Multivariable logistic regression analysis suggested that students are 3 times more likely to demonstrate problematic use compared with unemployed individuals (*P*=.03); adults using more than five apps are 2 times more likely to demonstrate problematic use compared to those using a maximum of three apps (*P*=.007). Protective factors against problematic smartphone use include using apps for academic (odds ratio [OR] 0.66; *P*=.04) or religious needs (OR 0.55; *P*=.007) and having a monthly family income of 5001-10,000 SAR (Saudi Riyal; US $1300-$2700; OR 0.46; *P*=.01) or 10,001-20,000 SAR (US $2700-$5400; OR 0.51; *P*=.03) compared to the <1501 SAR (US $400) income group.

**Conclusions:**

We reported a very high prevalence of problematic use of smartphones in KSA. Considering its negative impact on physical and psychosocial health, public health programs should develop preventive strategies.

## Introduction

Smartphones are considered the most used technological tool worldwide [1]. Smartphone addiction is a newly introduced term. The term is used by some due to the effects of overuse of smartphones on psychogenic illnesses and people’s social lives [2], or due to resulting urges and drives for repeated use, use in dangerous situations, dependence, tolerance, withdrawal symptoms, and interruptions to one’s work, social, and family life [3,4]. However, the conceptualization of smartphone overuse as an addiction remains controversial even among experts in this field [5]. Panova and Carbonell [2] argued that addiction is a disorder with severe effects on physical and psychological health; while a behavior such as overuse of a smartphone may have a similar presentation to addiction, that does not mean it should be considered an addiction. They propose moving away from the addiction framework when studying technological behaviors and using, instead, terms such as “problematic use” to describe them [2]. Nevertheless, excessive and problematic use of smartphone negatively impacts people’s lives, including their self-esteem [6]. The problematic use of a smartphone can be defined as “an inability to regulate one’s use of the mobile phone, which eventually involves negative consequences in daily life (e.g. financial problems)” [7].

One review of studies around the world found a mean problematic smartphone use prevalence of 18.9%, with a higher prevalence among women, and a trend of decreasing prevalence after the age of 20 [8]. Studies in the Kingdom of Saudi Arabia (KSA) have shown that about one third to half of the smartphone users exhibit problematic use [9-11]. Another local study suggests that the problematic use of smartphones is associated with negative effects on sleep, energy level, mood, eating habits, weight, exercise, and academic performance [12]. However, these studies were conducted with young adults; hence, they cannot be generalized to a wider population group. In fact, most global research projects have studied problematic smartphone use or smartphone addiction only among young people [13]. Additionally, no such studies have been conducted in the Qassim region of KSA.

In this context, this study aims to estimate the prevalence of problematic smartphone use among an adult population aged 18-65 years in the Qassim region of KSA. We also explored whether factors such as demographics, app use, and reason for app use were associated with the problematic use of smartphones.

## Methods

### Study Design and Settings

We conducted a cross-sectional survey of adult residents of the Qassim region of KSA. We recruited our participants from the Qassim University and primary health care centers (PHC) in the Qassim region. Qassim, officially known as the Emirate of Al-Qassim, is an administrative province of KSA. It is located in the northern central part of the Kingdom and has an estimated 1.02 million people living in 65,000 square kilometers [14].

### Recruitment

Male and female Saudi residents aged between 18 and 65 years were considered eligible for our study. We set an age cutoff due to limited access to residents older than 65 years. Individuals were excluded if they had any communicable respiratory illness or any other disease that made it difficult for them to participate in the study. We recruited participants from the Qassim University and PHC in the region using multistage cluster sampling. First, we developed a sampling frame comprising the primary sampling units—a list of Qassim University’s 15 colleges situated on the main campus and a list of all PHC (N=158) in Qassim. We randomly selected 6 colleges and 52 PHC from the list. We calculated our sample size using the Epi Info, version 7 (Centers for Disease Control and Prevention). For a probability value of .05 and 50% expected prevalence, we needed 384 participants from each group—university and PHC.

Data collectors visited the colleges over a period of 2 months to randomly enroll students for the study. To recruit adults from the general population, our data collector invited every third adult patient or visitor entering the selected primary health care centers during 3 consecutive days each week. Data collection continued over a period of 3 months (between December 2019 and February 2020). We ended data collection after completing 715 interviews because of the COVID-19 lockdown measures, of which 708 (99%) were considered for analysis. Participants’ characteristics are presented in Table 1.

**Table 1:** Sociodemographic characteristics of the participants (N=708).

Characteristics			Values
**Gender, n (%)**			
	Male	325 (45.9)	
	Female	383 (54.1)	
**Age range (years), n (%)**			
	18-24	518 (73.2)	
	25-34	114 (16.1)	
	≥35	76 (10.7)	
Mean, SD (years)			25.1 (8.5)
Median (years)			22.0
**Marital status, n (%)**			
	Single	553 (78.4)	
	Married	152 (21.6)	
**Education, n (%)**			
	Primary	12 (1.7)	
	Intermediate-secondary	511 (72.4)	
	Higher diploma	88 (12.5)	
	Bachelor or higher	95 (13.5)	
**Occupation, n (%)**			
	Unemployed	64 (9.1)	
	Student	515 (72.9)	
	Employed	127 (18.0)	
**Monthly family income, n (%)**			
	1500 SAR (US $400) or less	96 (14.2)	
	1501-5000 SAR (US $400-$1300)	97 (14.3)	
	5001-10,000 SAR (US $1300-$2700)	188 (27.8)	
	10,001-20,000 SAR (US $2700-$5400)	203 (30.0)	
	>20,000 SAR (>US $5400)	93 (13.7)	

### Procedures

The structured questionnaire included demographic information and the short version of the Smartphone Addiction Scale (SAS-SV) [15]. Demographic information included participants’ age, gender, educational level, marital status, current occupation, and income. The SAS-SV is a 10-item scale developed and validated in South Korea to measure smartphone addiction among adolescents [15]. Although we used the SAS-SV, we avoided the terminology “smartphone addiction” and used the terminology “problematic use of smartphones” instead, as explained in the introduction section.

Our questionnaire, including the SAS-SV, was translated into Arabic and reverse translated into English, and both were compared to ensure accuracy before starting data collection. Then, we carried out field testing with 24 Saudi adults to ensure that our questionnaire was understandable by our target population. The participants for field testing were purposively sampled to ensure diverse demographics for good representation of genders, income levels, education levels, and age groups. Field testing of the preliminary questionnaire was conducted by 2 male and 2 female medical students who were native Arabic speakers. Field testing was conducted in 3 phases of 8 interviews each, with the questionnaire undergoing revision after each phase. The final survey was conducted face-to-face by 6 male and 6 female final-year medical students who were trained to use the instrument.

### Ethics Approval

All researchers completed the ethics course recommended by the local institutional review board. We received ethics approval from the Institutional Review Board of the Ministry of Health, Qassim region, Saudi Arabia (Approval No. 1378136-1440). All study participants received a detailed informed consent document that explained the purposes of the study and highlighted the topics, types of questions, and the time involved in the study. Confidentiality and anonymity of all information collected from the participants were maintained, and the participants retained the right to refuse to answer specific questions or to opt out of the study at any time.

### Statistical Analysis

Data entry and analyses were carried out using the SPSS version 20 (IBM Corp). To classify problematic smartphone use, we first computed participants’ scores on each of the 10 SAS-SV items. Then, we used 31 and 33 as the male and female cutoff points, respectively, to determine problematic use [15]. We carried out descriptive analyses of sociodemographic and smartphone use characteristics, which were reported as percentages and frequencies. We conducted multivariable logistic regression analysis to investigate the factors associated with problematic smartphone use, reported as odds ratio (OR) with a 95% confidence interval. A *P* value of <.05 was considered statistically significant.

## Results

We interviewed 715 adults aged 18 to 65 years. However, 7 (1%) participants were dropped from further analysis due to incomplete information. Table 1 presents participants’ sociodemographic characteristics. Among the 708 participants, over half (n=383, 54%) were female; about three quarters (n=518, 73%) were aged between 18 and 24 years; over 78% (n=553) were single; 72.4% (n=511) had an intermediate-level education; 72.9% (n=515) were students; and 18% (n=127) were employed. Moreover, 193 (28.5%) participants had an average monthly family income of 5000 SAR (US $1300) or less, 188 (27.8%) had a monthly family income between 5001 SAR and 10,000 SAR (US $1300-$2700), while 203 (30%) had an income between 10,001 SAR and 20,000 SAR (US $2700-$5400).

**Figure 1:**
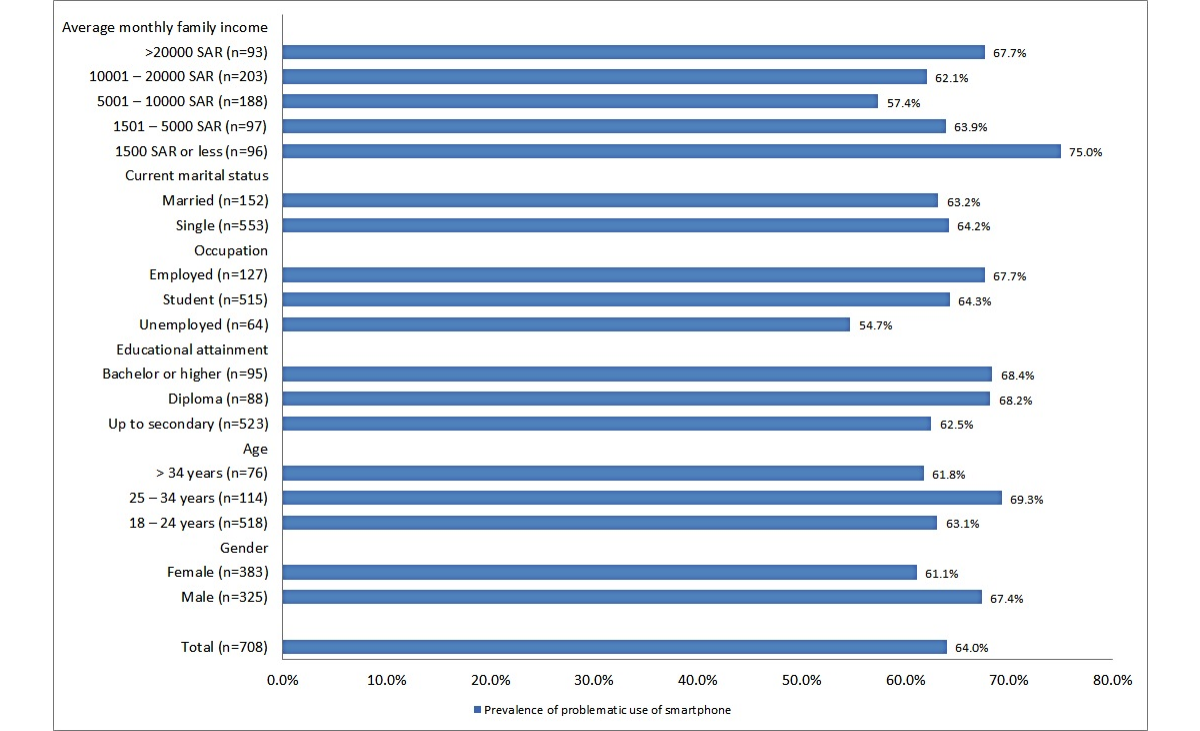
Prevalence of problematic use of smartphones among adults aged 18-65 years in Qassim, Kingdom of Saudi Arabia (cross-sectional survey, December 2019 to February 2020). SAR: Saudi Riyal.

Figure 1 presents the prevalence of problematic smartphone use in Qassim, KSA, by different sociodemographic groups. We estimated the overall prevalence at 64%. Among the income groups, the highest prevalence (n=96, 75%) was observed among the lowest monthly family income group (≤1500 SAR [US $400]). Prevalence among the single and married individuals was almost same (n=553, 63.2% and n=152, 64.2%, respectively). We observed a higher prevalence among employed adults (n=127, 67.7%) and students (n=515, 64.3%) compared with unemployed adults (n=64, 54.7%). Among the education groups, prevalence was lowest among the lowest education group (n=523, 62.5%) and highest among the highest education group (n=95, 68.4%). The prevalence of problematic smartphone use was higher among the 25-to-34-years age group (n=114, 69.3%) compared with the 18-to-24-years group (n=518, 63.1%) and the >34 years (n=76, 61.8%) age groups. Regarding gender, we found that men (n=325, 67.4%) had a higher prevalence of problematic smartphone use than women (n=383, 61.1%).

Table 2 presents characteristics of participants’ smartphone use. Almost all of them had been using a smartphone for more than 3 years. A quarter (172/688, 25%) of them were using up to three smartphone apps, while the rest were using more than three apps, with 28.6% (197/688) using six or more apps regularly. Our participants’ reasons for using smartphone apps included social networking (645/706, 91.4%), reading or listening to the news (424/706, 60.1%), watching movies or listening to music (392/706, 55.6%), academic/professional needs (260/706, 36.8%), searching for general knowledge (223/706, 31.6%), playing games (214/706, 30.3%), religious needs (176/706, 24.9%), and watching sports (157/706, 22.2%).

Table 3 presents the factors associated with problematic smartphone use among adults in KSA. Among the sociodemographic variables, no statistically significant association was found between problematic smartphone use and gender, age, marital status, or educational attainment. The multivariable logistic regression analysis suggests that students were 3 times more likely to have problematic smartphone use than the unemployed (OR 2.99; *P=*.03). However, no statistically significant difference was observed between the unemployed and employed groups (*P=*.22). Our results also suggest that compared with individuals with an average monthly family income of 1500 SAR (US $400) or less, those with an income of 5001 SAR to 10,000 SAR (US $1300-$2700) and 10,001 SAR to 20,000 SAR (US $2700-$5400) were 54% (OR 0.46; *P=*.01) and 49% (OR 0.51; *P=*.03) more likely to have problematic smartphone use, respectively. However, no significant difference was observed between the lowest (<1500 SAR [US $400]) and highest (>20,000 SAR [US $5400]) income groups (**P*=.50*).

Regarding characteristics of smartphone use, we found that compared with individuals who use 1 to 3 apps daily, users of more than 5 apps were 2 times more likely to have problematic smartphone use (OR 2.02; *P=*.007). Individuals who were using apps for academic or professional needs were 34% less likely to have problematic use (OR 0.66; *P=*.04), and individuals who were using the apps for religious purposes were 45% less likely to have problematic use (OR 0.55; *P*=.007) than those citing other reasons for use.

**Table 2:** Characteristics of participants’ smartphone use in Qassim, KSA^a^ (cross-sectional survey, December 2019-February 2020).

Smartphone use characteristics				Values, n (%)
**Duration of use (n=705)**				
	Up to 3 years		14 (2.0)	
	>3 years		691 (98)	
**Apps used on an average day (n=688)**				
	1-3 apps		172 (25)	
	4-5 apps		319 (46.4)	
	>5 apps		197 (28.6)	
**Use app notifications (n=690)**				
	No		123 (17.8)	
	Yes		567 (82.2)	
**Reason for using apps**				
	**To read or listen to news (n=706)**			
		No	282 (39.9)	
		Yes	424 (60.1)	
	**Social networking (n=706)**			
		No	61 (8.6)	
		Yes	645 (91.4)	
	**Academic or professional (n=706)**			
		No	446 (63.2)	
		Yes	260 (36.8)	
	**Playing games (n=706)**			
		No	492 (69.7)	
		Yes	214 (30.3)	
	**Watching sports (n=706)**			
		No	549 (77.8)	
		Yes	157 (22.2)	
	**General knowledge (n=706)**			
		No	483 (68.4)	
		Yes	223 (31.6)	
	**Religious (n=706)**			
		No	530 (75.1)	
		Yes	176 (24.9)	
	**Watching movies/music (n=706)**			
		No	314 (44.5)	
		Yes	392 (55.5)	

^a^KSA: Kingdom of Saud Arabia.

**Table 3:** Determinants of problematic smartphone use among adults (N=708) in Qassim, KSA^a^ (cross-sectional survey, December 2019-February 2020).

Determinant (reference category)				*P* value		Odds ratio^b^		95% CI for odds ratio		
								Lower		Upper
**Gender (male)**										
	Female			.55		0.89		0.61		1.30
**Age (18-24 years)**										
	25-34 years			.14		2.06		0.79		5.36
	>34 years			.22		1.98		0.67		5.87
**Education (up to intermediate or secondary)**										
	Higher diploma			.29		1.41		0.75		2.68
	Bachelor or above			.26		1.42		0.77		2.63
**Occupation (unemployed)**										
	Student			.03		2.99		1.14		7.86
	Employed			.22		1.61		0.75		3.45
**Current marital status (single)**										
	Married			.88		0.95		0.47		1.92
**Monthly family income (≤1500 SAR [US $400])**										
	1501-5000 SAR (US $400-$1300)			.27		0.68		0.35		1.34
	5001-10,000 SAR (US $1300-$2700)			.01		0.46		0.25		0.83
	10,001-20,000 SAR (US $2700-$5400)			.03		0.51		0.28		0.93
	>20,000 SAR (US $5400)			.50		0.79		0.40		1.57
**Use app notifications (no)**										
	Yes			.55		1.15		0.73		1.79
**Number of apps used in an average day (1-3 apps)**										
	4-5 apps			.13		1.41		0.91		2.20
	>5 apps			.007		2.02		1.21		3.35
**Reasons for using apps**										
	**Use apps to read or listen to news (no)**									
		Yes	.17		1.31		0.89		1.93	
	**Use apps for social networking (no)**									
		Yes	.24		1.47		0.78		2.77	
	**Use apps for academic or professional needs (no)**									
		Yes	.04		0.66		0.44		0.98	
	**Use apps to play games (no)**									
		Yes	.28		1.25		0.83		1.89	
	**Use apps to watch sports or games (no)**									
		Yes	.32		1.26		0.80		2.00	
	**Use apps for general knowledge improvement (no)**									
		Yes	.06		0.68		0.45		1.02	
	**Use apps for religious needs (no)**									
		Yes	.007		0.55		0.36		0.85	
	**Use apps to watch movies or listen to music (no)**									
		Yes	.88		1.03		0.69		1.54	

^a^KSA: Kingdom of Saud Arabia.

^b^Multivariable logistic regression analysis.

## Discussion

### Principal Findings

This study aimed to investigate the prevalence and associated factors of the problematic use of smartphones among adults aged 18-65 years in Qassim, KSA to reduce the gap in the literature. The majority of previous studies in this regard used exclusively college or university students [13]. We estimated a very high prevalence (64%) of problematic use of smartphone among this population groups. Determinants of the problematic use of smartphone include occupation, income, number of apps used, and reasons for using the apps.

We estimated the prevalence of problematic use of smartphones at 64% among adults aged 18 to 65 years in Qassim, KSA. This finding is in concordance with the findings reported by local studies conducted on university students, which were 71.9% [16] and 66% [10]. However, other local studies have shown smaller figures, for example 48% [17], 36.5% [11], and 19.1% [18]. A study that was conducted in 4 countries in the Middle East showed varying prevalence of problematic smartphone use: in Jordan, 59.8%; in KSA, 27.2%; in Sudan, 17.3%; and in Yemen, 8.6 % [19]. In other countries, studies have reported different figures: 38.9% in the United Kingdom [20], 38.5% in China [21], almost 30% in Malaysia [22], 21.5% in Belgium, and 12.5% in Spain [23].

Variation in prevalence could be affected by study design, sample size, or the scale used. Our study’s high prevalence could be explained by the fact that Saudi Arabia’s social media presence is one of the largest in the world. The large number of active social media users is mostly due to the high rate of smartphone ownership. With more than 84% of the population living in urbanized areas with very fast internet connections, it comes as no surprise that active social media users may number more than 25 million. According to reports from Hootsuite and We Are Social, Saudis are the largest group of active users on Instagram, Twitter, and Snapchat in the region [24].

Our results suggest that those with an average monthly family income of 5001 SAR (US $1300) to 20,000 SAR (US $5400) were less likely to have problematic smartphone use compared with people in the lower- or higher-income groups. In a study in China, the relationship of income with smartphone use was not clear [21]. However, a local Saudi study revealed a finding similar to ours and stated clearly that low-income individuals are more likely to have problematic smartphone use [17]. This is a difficult issue to explain. Could it be that poor people have fewer choices for entertainment or that lower-income students lack access to other information communication technologies [25]? Our participants with higher incomes also had a higher prevalence of problematic use. Zulkefly and Baharudin [26] concluded that students from higher-income families spent more time and money on their mobile phones.

Regarding characteristics related to smartphone use, we found that people using more than 5 apps were 2 times more likely to exhibit problematic smartphone use. A study in the United Kingdom showed that the use of social and communication apps significantly correlates with problematic smartphone use [27,28]. This could explain our finding because when using more than 5 apps, those apps will most likely include social media apps such as Snapchat and so on. In our study, we found that individuals who use apps for academic or professional or religious purposes were less likely to have problematic use.

In this study, there was no statistically significant association between problematic smartphone use and gender, age, marital status, or educational attainment. However, a multicenter study among Saudi university students showed that female students were more affected [18]. A study in Korea also reported that excessive use of smartphone and smartphone addiction–proneness is higher among females [6]. Furthermore, De-Sola Gutierrez et al [8] reported that all the studies included in their review indicated that women or girls have higher levels of dependence and problematic use than men or boys [8,29]. Our findings may differ because the older, married women included in our study were busy with other work, in contrast to the student groups who were the focus of many previous studies. We also used a higher problematic use cutoff point for women as suggested by the SAS-SV [15].

The relationship between marital status and problematic smartphone use is understudied as most previous research has focused on the young [8,13]. The only local study conducted among young adults (postgraduate medical residents) did not include marital status data [30].

With regard to age, other studies from different parts of the world have shown that the total time spent on cell phones decreases with age, with the highest times reported for people less than 20 years old. This is related to the decreased self-control found in this age group [8]. Our study did not include people younger than 18 years of age, but we found that students were 3 times more likely to have problematic smartphone use than the unemployed. One of the reasons for this high prevalence could be that educational material is now often available on the internet, and students may feel more comfortable using a smartphone to access them compared with using other devices.

In this study, there was no statistically significant difference between the unemployed and employed groups. Hence, time is seemingly not an issue for those with problematic smartphone use. In Spain, a study showed that unemployed individuals were more addicted to their smartphones than people in other employment categories [23], whereas in China, the relationship was not clear [21].

### Study Limitations

Our study had some limitations, mainly in data collection; we depended on self-reported data, which could be a source of bias. Another limitation was that the SAS-SV scale is not validated for use in this culture. A third limitation was our sampling technique; although we employed systematic random sampling to recruit study participants, accessing them only from PHC and one university in the Qassim region might have negatively affected representativeness.

### Conclusions

The overall prevalence of problematic smartphone use was high among our study participants, and this problematic use was associated with being a student and using more than 5 apps. An average monthly family income of 5001 SAR (US $1300) to 20,000 SAR (US $5400) and using apps for academic or professional and religious purposes were found to have a protective effect against problematic smartphone use. Our findings have implications for future public health programs in KSA. Considering the high prevalence of problematic smartphone use among adults and its negative impact on physical and psychosocial health, public health programs should develop and implement appropriate preventive strategies. Further studies should focus on investigating the association between health-related quality of life and problematic use of smartphones.

## Abbreviations

KSA: Kingdom of Saudi Arabia
OR: odds ratio
PHC: primary health care centers
SAR: Saudi Riyal
SAS-SV: Smartphone Addiction Scale–Short Version
